# ALKBH3 partner ASCC3 mediates P-body formation and selective clearance of MMS-induced 1-methyladenosine and 3-methylcytosine from mRNA

**DOI:** 10.1186/s12967-021-02948-6

**Published:** 2021-07-03

**Authors:** Kristian Lied Wollen, Lars Hagen, Cathrine B. Vågbø, Renana Rabe, Tobias S. Iveland, Per Arne Aas, Animesh Sharma, Bjørnar Sporsheim, Hilde O. Erlandsen, Vuk Palibrk, Magnar Bjørås, Davi M. Fonseca, Nima Mosammaparast, Geir Slupphaug

**Affiliations:** 1 Department of Clinical and Molecular Medicine, Norwegian University of Science and Technology, NTNU, 7491 Trondheim, Norway; 2 Clinic of Laboratory Medicine, St. Olavs Hospital, Trondheim, Norway; 3 PROMEC Core Facility for Proteomics and Modomics, Norwegian University of Science and Technology, NTNU, and the Central Norway Regional Health Authority Norway, Trondheim, Norway; 4 CMIC Cellular & Molecular Imaging Core Facility, Norwegian University of Science and Technology, NTNU, and the Central Norway Regional Health Authority Norway, Trondheim, Norway; 5 Department of Pathology and Immunology, Division of Laboratory and Genomic Medicine, Washington University School of Medicine, St Louis, MO 63110 USA

**Keywords:** Alkylating agents, ALKBH3, ASCC3, Epitranscriptome, 1-Methyladenosine, 3-Methylcytosine, 7-Methylguanosine, No-go decay, P-bodies, Ribosome quality control

## Abstract

**Background:**

Reversible enzymatic methylation of mammalian mRNA is widespread and serves crucial regulatory functions, but little is known to what degree chemical alkylators mediate overlapping modifications and whether cells distinguish aberrant from canonical methylations.

**Methods:**

Here we use quantitative mass spectrometry to determine the fate of chemically induced methylbases in the mRNA of human cells. Concomitant alteration in the mRNA binding proteome was analyzed by SILAC mass spectrometry.

**Results:**

MMS induced prominent direct mRNA methylations that were chemically identical to endogenous methylbases. Transient loss of 40S ribosomal proteins from isolated mRNA suggests that aberrant methylbases mediate arrested translational initiation and potentially also no-go decay of the affected mRNA. Four proteins (ASCC3, YTHDC2, TRIM25 and GEMIN5) displayed increased mRNA binding after MMS treatment. ASCC3 is a binding partner of the DNA/RNA demethylase ALKBH3 and was recently shown to promote disassembly of collided ribosomes as part of the ribosome quality control (RQC) trigger complex. We find that ASCC3-deficient cells display delayed removal of MMS-induced 1-methyladenosine (m^1^A) and 3-methylcytosine (m^3^C) from mRNA and impaired formation of MMS-induced P-bodies.

**Conclusions:**

Our findings conform to a model in which ASCC3-mediated disassembly of collided ribosomes allows demethylation of aberrant m^1^A and m^3^C by ALKBH3. Our findings constitute first evidence of selective sanitation of aberrant mRNA methylbases over their endogenous counterparts and warrant further studies on RNA-mediated effects of chemical alkylators commonly used in the clinic.

**Supplementary Information:**

The online version contains supplementary material available at 10.1186/s12967-021-02948-6.

## Background

Enzymatic modification of nitrogen bases in mRNA regulates processing, transport, lifetime and translation of mRNA molecules. In turn, this influences cell differentiation, stress responses, immunity, cognition and cancer development ([1–3] and references therein). To date, 11 endogenous base modifications have been described in mammalian mRNA. These are 7-methylguanosine (m^7^G, residing in the mRNA cap structure [4] as well as internally [5–7]), pseudouridine (Ψ) [8], inosine (I) [9], *N*4-acetylcytidine (ac^4^C) [10], 5-methylcytidine (m^5^C) [11], 5-hydroxymethylcytidine (hm^5^C) [12], 3-methylcytidine (m^3^C) [13], 1-methyladenosine (m^1^A) [14, 15], 6-methyladenosine (m^6^A) [16, 17] and its derivatives 6-hydroxymethyladenosine (hm^6^A) and 6-formyladenosine (f^6^A) [18]. Moreover, in silico analysis recently suggested a large number of potential 5-methyluridine (m^5^U) modification sites in mRNA [19]. Additional methylations at the ribose moieties further contribute to this complexity [2, 20]. Enzyme “writers” that are able to establish each of the base modifications have been described, and enzymes that catalyze their removal (“erasers”) have been described for the methylated bases except internal m^7^G. Proteins that specifically bind to and dictate the functional outcomes (“readers”) of the base modifications in mRNA are much less characterized, and are primarily confined to m^6^A, m^1^A and the m^7^G mRNA cap [21–27].

Notably, at least three of the endogenous mRNA modifications, m^1^A and m^3^C and m^7^G, may also be introduced in nucleic acids by direct non-enzymatic methylation by endogenous and environmental methylating agents, including tobacco-specific nitrosamines and agents frequently used as cytostatic drugs in cancer treatment [28, 29]. Although chemical methylation of human mRNA has previously not been specifically addressed, treatment of total RNA from different species with methyl methanesulfonate (MMS) or 1-methyl-nitrosourea (MNU) resulted in prominent formation of m^7^G as well as several fold higher relative levels of m^1^A and m^3^C in total RNA than in DNA [28]. m^1^A, m^3^C and m^7^G all introduce a positive charge in addition to the methyl group. This can dramatically affect RNA–protein interactions and secondary structures through electrostatic effects, and may interfere with mRNA–tRNA interactions [29–32]. A crucial question then is whether these aberrant methylations are recognized and processed differentially compared to their endogenous counterparts. We previously demonstrated that the human AlkB-homolog ALKBH3 is able to demethylate m^1^A and m^3^C in RNA as well as in DNA with single-strand substrate preference [33, 34]. Another member of the same family, ALKBH1, may contribute to demethylation of certain structured m^1^A sites in mRNA [35]. ALKBH3 is able to restore RNA function by reactivating chemically methylated RNA bacteriophages and tRNA and has been shown to act as an eraser of endogenous m^1^A in mRNA in vivo [15, 33]. Thousands of reversible m^1^A sites have been identified in mRNA from ALKBH3 knockout cells [15], whereas hundreds of sites have been identified in wild type cells [36]. Whether ALKBH3 shares a similar function in mRNA demethylation of endogenous m^3^C [13], remains unknown. The YTH-domain containing proteins YTHDF1-3 and YTHDC1, but not YTHDC2, have been reported to be m^1^A reader proteins [26, 37]. Presently, no readers are known for m^3^C or internal m^7^G in mRNA and no studies have attempted to monitor alterations in the human mRNA binding proteome after treatment with methylating agents.

To begin addressing these issues, we separately quantified endogenous and MMS-induced methylbases in HeLa mRNA after MMS treatment and monitored concomitant alterations in the mRNA binding proteome. Immediately after MMS treatment, we observed markedly increased levels of m^1^A, m^3^C and m^7^G in mRNA. This was accompanied by reduced binding of protein members of the ribosomal 40S subunit, whereas binding of 60S proteins was essentially unaffected. The selective 40S loss could be explained by ribosomal collisions at MMS-induced methylbases or secondary structures, thereby activating the ribosomal quality control (RQC) pathway (reviewed in [38]), GIGYF2/4EHP-dependent sequestration of the mRNA cap and blocked recruitment of 43S preinitiation complexes (PICs) to affected mRNAs [39, 40].

ASCC3 was among the very few proteins that increased binding to mRNA after MMS treatment. ASCC3 is a DNA helicase that associates with ALKBH3 to facilitate unwinding and efficient demethylation of m^1^A and m^3^C in DNA [41, 42]. In addition, it was recently identified to bind stalled ribosomes as part of the RQC trigger (RQT) complex that facilitates ribosomal splitting [43]. Our results suggest that cells exploit these properties of ASCC3 to allow ALKBH3-mediated demethylation of aberrant m^1^A and m^3^C in the coding region of mRNA, whereas canonical methylbases remain unaffected. In addition, ASCC3 has a role in P-body formation, potentially by promoting ribosomal detachment from mRNAs harboring difficult-to-repair lesions and assembly of such transcripts in P-bodies for further repair or degradation. Although the molecular details of such a mechanism remain to be elucidated, our results provide first evidence of selective removal of aberrant methylbases from the human mRNA pool and may have significant implications to modulate the effects of cytotoxic alkylating drugs.

## Methods

### Chemicals, plasmids and antibodies

Complete protease inhibitor cocktail was from Roche Inc. Dulbecco’s modified Eagle medium (DMEM) minus l-arginine and l-lysine, dialyzed fetal calf serum (dFCS) and trypsin were from Thermo Scientific. Stably isotope labeled l-arginine and l-lysine were from Cambridge Isotope Laboratories. All other chemicals were from Sigma Aldrich. Full-length ASCC3 with a C-terminal HA-tag and CFP-DCP1A have been described previously [44, 45].

Primary antibodies and dilutions: ALKBH3 (Santa Cruz, sc-376520, IF: 1/100, WB: 1/2000), ASCC3 (Atlas, HPA001439, IF: 1/50), *β*-actin (Abcam, ab8226, IB: 1/2000), CELF1 (Santa Cruz, SC-20003, IB: 1/1000, IF: 1/200), EIF2S1 (Atlas, HPA064885, IB: 1/1000), EIF2S1-pS51 [Abcam, ab32157, IB: 1/500. Note that in the canonical human EIF2S1 sequence, this serine is at position 52 (UniProtKB-P05192)], HA-tag (Santa Cruz, SC-805, IF: 1/100). Histone H3 (Abcam, ab1791, IB: 1/2000), HNRNPA1 (Santa Cruz Biotechnology, SC-32301, IB: 1/3000, IF: 1/200), RPS10 (Atlas, HPA048084, IB: 1:125), RPL18 (Atlas, HPA046572, IB: 1:250), SERBP1 (Sigma, WH0026135M1, IB: 1/1000, IF: 1/50), SND1 (Sigma, HPA002632, IB: 1/1000, IF: 1/50), TIA1 (Abcam, ab196382, IF: 1/100), HRP-conjugated swine anti-rabbit (P0399) and rabbit anti-mouse (P0260) secondary antibodies were from Dako Chemicals. IRDye® 680RD Goat anti-Mouse IgG and IRDye® 680RD Goat anti-Rabbit IgG secondary antibodies were from LiCor Biotechnology. Secondary antibodies for confocal microscopy were from Life Technologies; Alexa Fluor® 488 rabbit anti-mouse (#A-11059) and donkey anti-rabbit (#A-21206), Alexa Fluor® 532 goat anti-mouse (#A-11002) and goat anti-rabbit (#A-11009), Alexa Fluor® 647 donkey anti-goat (#A-21477) and goat anti-rabbit (#A-21244).

### Cell culture

HeLa S3 cells were from ATCC. PC-3 WT and ASCC3 knockout cells were from the Washington University Genome Engineering and iPSC Center (GEiC) and the knockout cells generated as described [42]. Primary stocks were employed subsequent to amplification [41]. Cells were grown in 5% CO_2_ at 37 °C in DMEM supplemented with 10% fetal calf serum, 0.1 mg/ml gentamicin, 2.5 µg/ml amphotericin B and 0.03% l-glutamine. All cell lines were mycoplasma free as determined by LC–MS/MS peptide analysis. For Stable Isotope Labeling with Amino acids in cell culture (SILAC) experiments, HeLa cells were grown as above, but using DMEM minus l-arginine and l-lysine, and dFCS. For labeling of light, medium and heavy HeLa cells, l-lysine-^12^C_6_/l-arginine-^12^C_6_, l-lysine-^2^H_4_/l-arginine-^13^C_6_ and l-lysine-^15^N_2_^13^C_6_/l-arginine-^15^N_4_^13^C_6_ were added to the media, respectively. To increase sensitivity of the MS analysis, we reduced the number of target peptides resulting from arginine-to-proline conversion by reducing the amount of l-arginine (and l-lysine) in the growth media as previously described [46, 47]. Cells were transfected with Fugene HD® (Roche) according to the manufacturer’s instructions and analyzed 24 h later. CRISPR–Cas9-mediated editing of ASCC3 in PC-3 was described previously [42].

### Cell cycle analysis and resazurin reduction assay

Flow cytometric analysis was performed as previously described [48]. Briefly, cells were fixed in methanol (70%), RNase treated (100 µg/ml in PBS, 37 °C, 30 min) and propidium iodide stained (50 µg/ml in PBS, 37 °C, 30 min) prior to analysis by FACS flow cytometry (FASCAria, BD Bioscience). 24 h after MMS treatment, 20 µl 2.5 mM resazurin, PBS was added to each well of a 96-well plate containing 100 µl cell growth medium/well and incubated further for 2 h. Resorufin fluorescence was subsequently determined using a microplate reader (BMG Labtech Fluostar Omega, Oslo, Norway, excitation 544 nm, emission 590 nm).

### MMS treatment, UV cross-linking and oligo(dT) capture

Oligo(dT) capture of mRNA was performed as previously described [49] with minor modifications. For each condition, 2 × (for western analysis) or 4 × (for MS analysis) 15 cm cell culture dishes were used, and the volumes of buffers were equally reduced according to cell growth area compared to the original protocol. MMS was added to the growth medium at a final concentration of 1 mM, plates covered with Parafilm and cells incubated further for 1 h. After a brief wash with pre-warmed PBS, new medium was added, and the cells incubated further for various times prior to analysis. After medium removal, culture plates (60–80% confluence) were placed on ice, washed 2 × with ice cold PBS prior to UVC irradiation (254 nm) in a UV Stratalinker® 2400. To reduce the amount of starting material in the SILAC approach, we first optimized the published protocol [49] with respect to the UV dose. A reduced dose (25 mJ/cm^2^ UVC) mediated the same extent of cross-linking as higher dosages while also reducing the tendency of beads sticking to the surfaces of plastic tubes and pipette tips, thus improving capture of mRNA binding proteins (Additional file 1: Figure S1A). Comparison of oligo(dT)-captured samples from UVC-irradiated versus non-irradiated cells by SDS-PAGE and SimplyBlue™ staining confirmed the specificity of the protocol (Additional file 1: Figure S1B). After UV cross-linking at 25 mJ/cm^2^, cells were harvested, added lysis buffer (20 mM Tris–HCl pH 7.5, 0.5 M LiCl, 1% lithium dodecyl sulphate (LDS, w/v), 5 mM EDTA, 5 mM DTT, 1 × Complete protease inhibitor cocktail) and homogenized by passing the lysates several times through a syringe with different needles (2 × 23G, 2 × 25G and 3 × 27G) and the homogenates snap-frozen in liquid N_2_. After thawing, samples were clarified by centrifugation at 12,000×*g* and protein concentration measured by NanoDrop spectrophotometry. For SILAC experiments, equal amounts of protein from control (PBS), noUV and 0 h MMS extract, or control, 4 h MMS and 15 h MMS extract were mixed and subjected to oligo(dT) capture. Poly(A)-containing RNAs and cross-linked proteins were captured using oligo(dT)_25_ magnetic beads (New England Biolabs). Beads were then washed 3 × with 4.5 ml buffer 1 (20 mM Tris–HCl pH 7.5, 0.5 M LiCl, 0.5% LDS (w/v), 5 mM EDTA, 1 mM DTT) and then 1 × with 4.5 ml buffer 2 [20 mM Tris–HCl pH 7.5, 0.2 M LiCl, 0.5% NP-40 (v/v), 1 mM EDTA, 1 mM DTT]. To avoid LiCl/LDS precipitation, steps including lysis buffer and buffer 1/2 were performed at room temperature (RT). Beads were eluted with elution buffer (20 mM Tris–HCl pH 7.5, 1 mM EDTA) for 3.5 min at 70 °C. The entire oligo(dT) capture procedure was then repeated 2 × with the same extract and magnetic beads. Eluates were pooled and treated with RNase T_1_ and RNase A (Sigma), concentrated using Amicon Ultra 0.5 ml Filters (3 kDa cut-off, Millipore) and separated by PAGE as described below.

### Electrophoresis and western analysis

Proteins were denatured in 1 × LDS sample buffer (Invitrogen) at 70 °C for 10 min and then electrophoresed in 10% Novex Bis–Tris gels using MOPS running buffer. Gels were then either stained (Simply Blue™ Safe Stain, Invitrogen) or electroblotted onto PVDF-membranes (XCell II™ Blot Module, Immobilon®-P^SQ^) according to the manufacturer’s instructions. Blots were blocked in PBST (phosphate-buffered saline, 0.1% Tween 20), 5% fat-free dry milk for 1 h, and then incubated for 1 h with primary antibody in PBST, 1% fat-free dry-milk. After 3 × 5 min washes in PBST, membranes were incubated for 1 h in secondary-HRP-conjugated antibody (1/5000) in PBST, 1% fat-free dry-milk (for antibodies recognizing phosphorylated proteins PBST was replaced by TBST). Subsequent to 3 × 5 min washes in PBST/TBST, membranes were developed using SuperSignal West Femto (Pierce) and visualized using a Kodak Image Station 4000R and images analyzed using Kodak molecular imaging software 4.0. When IRDye® secondary antibodies were used, the membranes were visualized using Odyssey Imager (LiCor) and images analyzed using Image Studio 3.1.

### Protein mass spectrometry and data analysis

To analyze overall proteome changes subsequent to MMS treatment, 100 µg of each of the mixed SILAC extracts (pre-oligo(dT) capture) were precipitated using chloroform/methanol [50] and separated by LDS-PAGE as described above in parallel with the mRNA-binding protein (mRBP) samples. Each total protein lane was cut into 5 slices, while each of the two mRBP enriched lanes were cut into 3 slices. Proteins in each slice were then reduced and alkylated prior to in-gel trypsination as described [51]. Peptides were resuspended in 0.1% formic acid and analyzed by LC–MS/MS using an EASY-nLC 1000 UHPLC system (Thermo Scientific/Proxeon) coupled to an LTQ-Orbitrap Elite hybrid mass spectrometer operating in positive ion- and data dependent acquisition mode. Peptides were injected onto a C-18 trap column (Acclaim PepMap100, 75 μm i. d. × 2 cm, C18, 5 μm, 100 Å, Thermo Scientific) and further separated on a C-18 analytical column (Acclaim PepMap100, 75 μm i. d. × 50 cm, C18, 3 μm, 100 Å, Thermo Scientific) using either 120 min (oligo(dT) eluates) or 180 min (total protein) multi-step gradients from 2 to 40% CH_3_CN in 0.1% formic acid at a flow rate of 250 nl/min. The following parameters were used for the LTQ-Orbitrap Elite hybrid mass spectrometer: electrospray voltage 2.2 kV, CID fragmentation with normalized collision energy 35, automatic gain control (AGC) target value of 1E6 for Orbitrap MS1 and 1E3 for MS2 scans. Each MS1 scan (m/z 400–16,000) was acquired at a resolution of 120,000 FWHM, followed by 20 MS2 scans triggered for intensities above 500, at a maximum ion injection time of 200 ms for MS1, and 50 ms (total protein) or 120 ms (oligo(dT) eluates) for MS2 scans.

Raw files from the three independent biological replicates were analyzed in Proteome Discoverer 2.0 (Thermo Scientific) using Mascot version 2.2.06 with Uniprot database from Jan 2014 and Sequest HT bundled with Proteome Discoverer version 2.0.0.802 using Uniprot database from Apr 2015 for Homo sapiens [52]. The following search parameters were used: enzyme specified as trypsin with a maximum of two missed cleavages; precursor mass tolerance 10 ppm and fragment mass tolerance 0.6 Da. Carbamidomethyl (C) was set as fixed modification, while N-terminal acetylation, methionine oxidation, phosphorylation (S, T, Y) and isotope labeled l-lysines and l-arginines were set as dynamic modifications. The Percolator tool was used for peptide validation using a cutoff value of 0.01 for false discovery rate (FDR), and thus only peptides with a high confidence were used for final protein identification and quantification. Statistical analysis was performed using Perseus 1.5 [53]. SILAC ratios were normalized by protein median and log_2_ transformed. Proteins with a ratio for noUV/PBS and not showing an enrichment of at least twofold (non-normalized log_2_ noUV/PBS ≤ 1) in at least one biological replicate were filtered out, and proteins with an inconsistent directionality of SILAC ratios in label swap experiments were also excluded. The list was further filtered to only contain proteins quantified with at least six out of nine ratios (0 h, 4 h and 15 h). The three time points (0 h, 4 h and 15 h) were subjected to one-way ANOVA analysis using Benjamini–Hochberg FDR correction [54]. As an altered protein ratio in the oligo(dT) eluates might be caused either by altered mRNA binding of the protein or altered protein amount in the input extracts, we performed a pairwise t-test between oligo(dT) eluates and input extracts for each time point. Correcting for multiple testing was performed as above. ANOVA and t-test results were only considered significant if corrected p-value < 0.05 and median log_2_ SILAC-ratio/t-test difference less than − 0.5 or more than 0.5.

### LC–MS/MS quantification of methylated nucleosides

RNA samples were digested by incubation with 0.2 U nuclease P1 (Sigma, N8630), 20 U benzonase (Santa Cruz Biotech, SC-391121B) and 0.1 U alkaline phosphatase (Sigma, P5931) in a buffer containing NH_4_Ac pH 6.0 and 1 mM MgCl_2_ at 40 °C for 40 min. The hydrolysates were added three volumes of ice-cold acetonitrile and centrifuged at 16,000×*g* for 30 min at 4 °C. Supernatants were dried and dissolved in 50 µl water for LC–MS/MS analysis of methylated nucleosides. A portion of each sample was diluted for LC–MS/MS analysis of unmodified nucleosides. Chromatographic separation was performed using an Agilent 1290 Infinity II UHPLC system with an ZORBAX RRHD Eclipse Plus C18 150 × 2.1 mm ID (1.8 μm) column protected with an ZORBAX RRHD Eclipse Plus C18 5 × 2.1 mm ID (1.8 µm) guard column (Agilent). The mobile phase consisted of water and methanol (both added 0.1% formic acid) run at 0.3 ml/min for methylated nucleosides starting with a 6-min gradient of 5–90% methanol, followed by 3 min re-equilibration with 5% methanol, and for unmodified nucleosides maintained isocratically with 20% methanol. Mass spectrometry detection was performed using an Agilent 6495 Triple Quadrupole system operating in positive electrospray ionization mode, monitoring the mass transitions 282.1/150.1 (m^1^A, m^6^A), 285.1/153.1 (D_3_-m^1^A, D_3_-m^6^A), 258.1/126.1 (m^3^C, m^5^C), 261.1/129.1 (D_3_-m^3^C, D_3_-m^5^C), 298.1/166.1 (m^7^G, m^6^G), 301.1/169.1 (D_3_-m^7^G, D_3_-m^6^G), 268.1/136.1 (A), 244.1/112.1 (C), 284.1/152.1 (G), 245.1/113.1 (U), 266.1/150.1 (m^1^dA), 252.1/136.1 (dA), 228.1/112.1 (dC), 268.1/152.1 (dG), and 243.1/127.1 (dT). Quantification was performed by comparison with pure nucleoside standards.

### Confocal microscopy and image analysis

Cells were examined using a Zeiss LSM 510 or a Leica SP8 laser scanning microscope equipped with a Plan-Apochromat 63 × /1.4 oil immersion objective. DAPI was excited at 405 nm and detected at 420–480 nm. CFP was excited at 458 nm and detected using a 545 secondary beam splitter combined with LP 475. Alexa Fluor® 488 was excited at 488 nm and detected at 505–530 nm. Alexa Fluor® 532 and 555 were excited at 543 nm and detected at 560–615 nm. Alexa Fluor® 647 was excited at 633 nm and detected above 650 nm. Optical section thickness was kept constant for all channels and a pinhole diameter of 1 Airy unit was used for the 633 nm excitation wavelength to keep high signal-to-noise ratio in all channels.

Cells were grown on no. 1.5 glass coverslips (Marienfeld) in phenol red-free growth media and washed with ice-cold PBS before fixation in 4% paraformaldehyde for 15 min at RT and permeabilization with 0.1% Triton-X 100, PBST for 15 min at RT. Cells were then washed 3× with PBST and blocked in 1% newborn goat serum (NGS), PBST for 45 min at RT. Staining was performed overnight at 4 °C with the primary antibodies at 20 µg/ml in 0.5% NGS in PBST. Coverslips were washed 4× in PBST and stained with Alexa Fluor® secondary antibodies (1:400 in PBST) for 2 h at room temperature. Coverslips were mounted using Vectashield® mounting medium with DAPI or SlowFade Antifade Diamond mounting medium without DAPI (Thermo Scientific). Since nuclear staining was not feasible when three differentially stained proteins were analyzed, nuclei and cytoplasm were confidently identified by intermittent differential interference contrast (DIC). Images were analyzed in Imaris, version 8.2.0. The sample size [10] to estimate the number of P-bodies per cell, as well as the size of the P-bodies in wt vs. ASCC3 knockdown cells was chosen by estimating a mean reduction in difference by 50%, a standard deviation of 40% of the mean, a statistical power of the test of 80%, and a type 1 error of 0.05. The sample size to determine the fraction of cells positive for P-bodies (> 25) was chosen by roughly estimating 60% for the wt and 20% for the knockdown cells, based on previous observations, a power of 80% and a type-1 error of 0.05 [55].

## Results

### MMS mediates direct induction of methylbases that are also present endogenously in human mRNA

Several studies have quantified levels of various methylated RNA bases subsequent to treatment with alkylating agents ([28, 56] and references therein). However, to the best of our knowledge, no studies have specifically monitored chemical methylation in human mRNA. To determine a relevant and non-lethal dose of MMS, HeLa cells were first treated for 1 h with increasing doses of MMS and cell viability analyzed 24 h later. Based on the data, we chose to treat cells with 1 mM MMS for 1 h, mediating a relative survival of 90% (Additional file 2: Figure S2A) but with a clear S-phase delay around 4 h after MMS treatment (Additional file 2: Figure S2B). 24 h after treatment, cells had returned to essentially normal asynchronous distribution. To quantify the levels of methylated RNA bases, total RNA was extracted from non-treated and MMS-treated HeLa cells. mRNA was enriched by oligo(dT) capture from the same samples and global modification levels in total- and mRNA were quantified by LC–MS/MS. Although oligo(dT) captures both mRNAs and other polyadenylated RNA species like long non-coding RNAs (lncRNAs), about 90% generally constitutes protein-coding RNA [57].

**Fig. 1:**
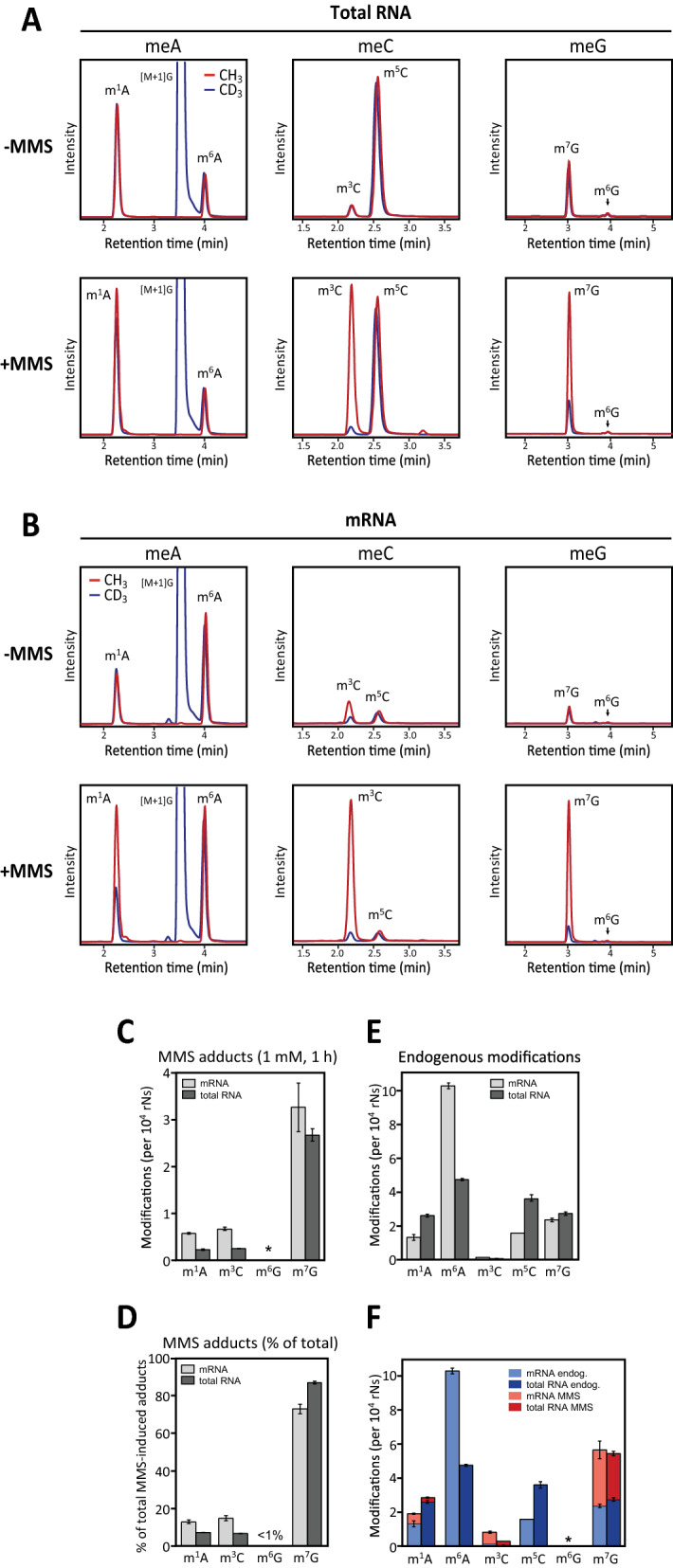
Endogenous and MMS-induced base methylations in total- and mRNA in HeLa cells. **A** Mass chromatograms of methylated bases in total RNA isolated from non-treated (upper panels) or MMS-treated (lower panels) HeLa cells after 1 h exposure of 1 mM MMS. Red graphs represent fraction of the bases containing light (CH_3_) methyl and blue graphs represent fraction containing deuterated (CD_3_) methyl in each modified nucleoside. Increased CH_3_/CD_3_ ratios after MMS treatment are mediated by non-enzymatic, MMS-mediated methylation (CH_3_). **B** Similar as in **A**, but with mRNA. **C** Concentrations of MMS-induced methyl adducts in total- and mRNA. **D** Relative distribution of MMS-induced methyl adducts in total- and mRNA. **E** Endogenous levels of methylated bases in total- and mRNA in untreated cells. **F** Total number of various base methylations in total- and mRNA with the relative amounts of endogenous and MMS-induced base methylations as indicated by differential coloring. Each bar in C-F represents the mean of three biological replicates with SDs as indicated

To individually monitor endogenous (enzymatic) and MMS-induced (non-enzymatic) methylation, cells were cultured for > 10 generations in medium containing a 1:1 mixture of non-labelled and deuterated methionine prior to MMS treatment. Within cells, methionine is converted to S-adenosyl methionine (SAM), the only known methyl donor for enzymatic RNA methylation. Thus, enzymatic methylation alone will mediate essentially equal amounts of light and heavy methylated ribonucleosides whereas non-enzymatic MMS-induced methylation will mediate increased CH_3_/CD_3_ ratios in the methylated ribonucleosides. Mass chromatograms from total- and mRNA analyses are illustrated in Fig. 1A, B. The relative quantities of each modification in untreated and MMS-treated cells are illustrated in Fig. 1C, D. Indeed, essentially identical levels of CH_3_ and CD_3_ were observed for all five methylnucleosides quantified in total RNA in the absence of MMS treatment (Fig. 1A, upper panel). MMS treatment resulted in markedly increased CH_3_/CD_3_ ratios in m^1^A, m^3^C and m^7^G, whereas the CH_3_/CD_3_ ratios in m^5^C and m^6^A remained essentially unchanged (Fig. 1A, lower panel), consistent with the notion that MMS does not induce these lesions [28].

In poly(A)RNA from untreated cells (Fig. 1E), m^6^A was found to be the dominant endogenous base methylation, in agreement with previous reports [58, 59]. Moreover, substantial amounts of m^1^A, m^7^G, m^5^C and m^3^C were also detected. Whereas m^6^A was twofold enriched upon oligo(dT) capture, m^1^A was correspondingly depleted. This verifies the successful enrichment of mRNA, since m^1^A is more abundant in the rRNA and tRNA pools than in mRNA [60, 61], whereas m^6^A is most abundant in mRNA [62]. Nearly identical levels of deuterated and non-deuterated methyl groups were observed for all the methylbases except m^3^C, which displayed a markedly higher CH_3_/CD_3_ ratio (Fig. 1B, upper panel). The cause of this is unknown, but could indicate a kinetic isotope effect or, more likely, isotope exchange catalyzed by the m^3^C methyltransferase METTL8 [13] or a yet unidentified m^3^C methyltransferase. Notably, SAM-independent hydrogen isotope exchange has previously been reported for tRNA m^5^U methyltransferase [63].

MMS treatment mediated markedly increased CH_3_/CD_3_ ratios in m^1^A, m^3^C and m^7^G, (Fig. 1B, lower panel). Levels of the different MMS-adducts (per 10^4^ unmodified ribonucleosides) in total- and mRNA are shown in Fig. 1C and conform well to those previously reported in total RNA subsequent to MMS treatment [28, 56]. The levels of endogenous methylations in untreated cells are shown in Fig. 1E, F illustrates the sum of each endogenous and MMS-induced methylation in total- and mRNA. MMS induced higher levels of the methyl adducts in mRNA compared to total RNA. This was especially evident for m^1^A and m^3^C, which were induced at more than twofold higher density in mRNA relative to total RNA (Fig. 1C).

**Fig. 2:**
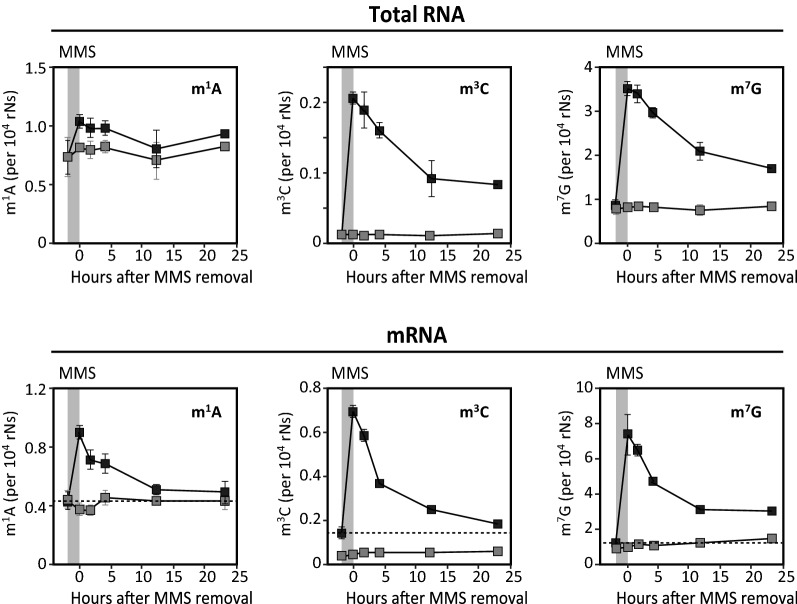
Kinetics of removal of endogenous and MMS-induced m^1^A, m^3^C and m^7^G from total-and mRNA subsequent to 1 h exposure of HeLa cells to 1 mM MMS. Grey curves: endogenous (CD_3_) modifications; Black curves: aberrant, MMS-induced (CH3) + endogenous (CH_3_) modifications. Grey rectangle: period of MMS treatment. Dashed line: Baseline MMS-induced lesions

We then monitored reversal of m^1^A, m^3^C and m^7^G in total- and mRNA at various time points after 1 h MMS treatment (Fig. 2). Upon MMS removal, a gradual reduction of all three lesions in mRNA followed essentially first order kinetics. Notably, whereas m^1^A and m^3^C returned to near pre-treatment levels after 24 h (~ 10% MMS-induced lesions remaining), about 30% residual MMS-induced m^7^G adducts were still present. m^1^A and m^3^C are both substrates for ALKBH3, but no human RNA m^7^G demethylase is known. Thus, removal of MMS-induced m^7^G is likely mediated by mRNA degradation only, whereas removal of m^1^A and m^3^C may be mediated by combined mRNA degradation and ALKBH3-mediated demethylation. Moreover, removal of all three methylbases was confined to the MMS-induced lesions, whereas the corresponding endogenous methylbases remained essentially unaffected. This strongly suggests that the cells harbor mechanisms to specifically recognize and remove aberrant methylbases from mRNA.

### MMS-induced changes in the mRNA binding proteome

To identify proteins differentially binding to mRNA at various time points after MMS treatment, we adapted a previously published UV cross-linking protocol [49] (Additional file 3: Figure S3A) combined with a triple-SILAC mass spectrometry (MS) approach [64] (Additional file 3: Figure S3B). By combining two time-course experiments using a common control (PBS treatment), a four time-point profile of the changes was achieved: (i) prior to MMS treatment (PBS), (ii) immediately after 1 h MMS treatment (0 h), (iii) 4 h and (iv) 15 h after end of MMS treatment (Additional file 3: Figure S3B). We also included a control that was neither MMS-treated nor UVC cross-linked (no UV). After covalent cross-linking by UVC irradiation at an optimized dose of 25 mJ/cm^2^ (Additional file 1: Figures S1A,B), cells were lysed and equal amounts of protein extract from the three cell populations were mixed (no UV/PBS/0 h and PBS/4 h MMS/15 h MMS). mRNA was enriched using oligo(dT) magnetic beads and mRNA binding proteins as well as proteins in the extracts were quantified using Orbitrap Elite LC–MS/MS. After filtering of three biological replicates, 336 proteins were quantified with at least six out of nine ratios (Additional file 4: Table S1). All of these proteins have previously been reported to be RNA-binding proteins (RBPs) [65, 66], underscoring the specificity of our experimental approach. In a previous study by Boucas et al. of proteins differentially binding to mouse embryonic fibroblast (MEF) mRNA subsequent to etoposide treatment [67], 184 proteins were quantified in both non-treated and treated cells. Out of these, 127 were also identified in our dataset (Additional file 3: Figure S3C), suggesting that the mRNA surveillance machinery is highly conserved between the species.

**Fig. 3:**
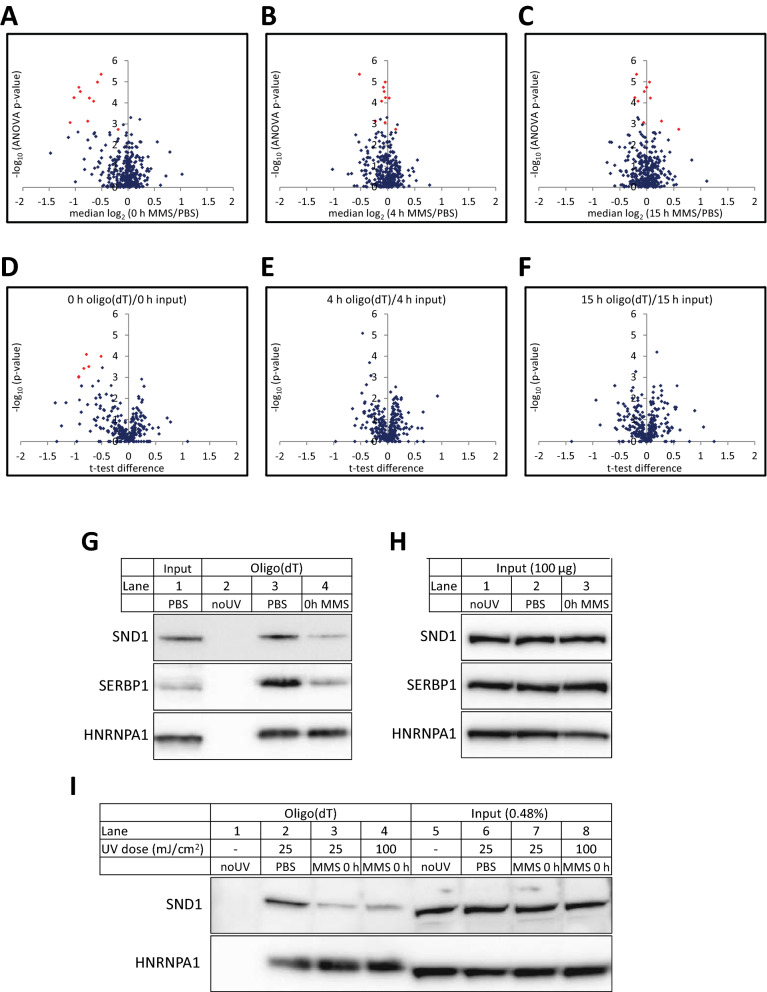
Temporal changes in the mRNA binding proteome after MMS treatment. **A**–**C** ANOVA p-values (−log_10_) plotted against median SILAC ratios (log_2_) at 0 h (**A**), 4 h (**B**) or 15 h (**C**). **D**–**F** Volcano plots showing t-test p-values (−log_10_) versus median SILAC ratios (log_2_) of oligo(dT)/input extract at 0 h (**D**), 4 h (**E**) and 15 h (**F**). Significantly altered proteins after Benjamini Hochberg FDR correction (< 0.05) are shown in red. **G**-**I** Verification of SILAC data by western analysis. HeLa cells were treated as indicated with either PBS (control) or MMS for 1 h, lysed and subjected to oligo(dT) enrichment. **G** SND1 and SERBP1 show reduced binding to m RNA, while HNRNPA1 does not alter its binding. **H** Input of extracts utilized for oligo(dT) enrichment in **G**. Note that the protein levels in lanes 1–3 remain the same. **I** Reduced mRNA binding is not caused by cross-linking bias. As in **G**, but MMS-treated cells were irradiated with either 25 mJ/cm^2^ (standard) or 100 mJ/cm^2^ (4 × standard dose). Note that increasing the UVC dose does not lead to increased cross-linking (lane 3 vs. lane 4)

Figure 3A-C show the log_2_ SILAC ratios at the various time points after MMS treatment plotted against the corresponding ANOVA p-value. Differentially expressed proteins (Benjamini Hochberg corrected p-values < 0.05, absolute median log_2_ SILAC-ratio > 0.5) observed in at least one time point are highlighted in red in the plots and provided in Table 1 together with additional selected proteins. The log_2_ SILAC ratios revealed very few and small alterations, and nearly all the differentially bound proteins showed reduced binding to mRNA after MMS treatment. Furthermore, very few RBPs showed significantly altered log_2_ ratios except immediately after 1 h MMS treatment (0 h). As changes in the SILAC ratios could be due to either altered mRNA binding or alterations of the protein levels in the input extracts induced by MMS treatment, we subjected the SILAC ratios of the input extracts to a one-way ANOVA analysis. None of the oligo(dT)-enriched proteins showed significantly altered amounts in the input extracts (Additional file 4: Table S1). We also performed pairwise t-tests between oligo(dT)-enriched samples and the corresponding input extracts, shown in Fig. 3D–F. Here, 6 proteins at 0 h (Fig. 3D), while no proteins at 4 h (Fig. 3E) and 15 h (Fig. 3F) were significantly altered between oligo(dT) eluates and input extracts. Thus, MMS treatment mediated rapid and transient loss of a small subset of RBPs from the enriched mRNA.

**Table 1:** Proteins displaying most affected binding to mRNA subsequent to MMS treatment

Gene	Protein name	Anova	t-test significance			Median log_2_ SILAC ratios		
			0 h	4 h	15 h	0 h	4 h	15 h
Significant differential binding								
ZC3HAV1	Zinc-finger CCCH-type antiviral protein 1	+	+			− 0.51	− 0.52	− 0.19
SND1	Staphylococcal nuclease domain-containing protein 1	+	+			− 1.03	− 0.04	− 0.22
HDLBP	Vigilin	+				− 0.65	− 0.11	− 0.16
ZC3H8	Zinc finger CCCH domain-containing protein 8	+				− 0.77	− 0.23	0.27
SERBP1	Plasminogen activator inhibitor 1 RNA-binding protein	+	+			− 1.10	− 0.05	− 0.06
NSUN2	tRNA (Cytosine(34)-C(5))-methyltransferase	+			+	− 0.19	0.15	0.59
RPSA	40S ribosomal protein SA	+	+			− 0.58	− 0.05	0.05
RPS3	40S ribosomal protein 3	+	+			− 0.93	− 0.08	0.00
RPS10	40S ribosomal protein 10	+				− 0.74	0.03	0.07
RPS14	40S ribosomal protein 14	+	+			− 0.91	− 0.07	− 0.05
RPS26P11	Putative 40S ribosomal protein S26-like 1		+			− 0.81	− 0.08	0.05
Sub-significantly increased binding								
ASCC3	Activating signal cointegrator 1 complex subunit 3					0.72	− 0.02	0.21
YTHDC2	Probable ATP-dependent RNA helicase YTHDC2					0.56	0.03	− 0.01
TRIM25	E3 ubiquitin/ISG15 ligase TRIM25					0.80	0.05	0.06
GEMIN5	Gem-associated protein 5					0.62	0.78	0.50

To further validate our data, the levels of selected proteins were also monitored by Western blot analysis. Here, SND1 and SERBP1 displayed reduced binding to mRNA immediately after MMS treatment, whereas no change was observed for HNRNPA1 (Fig. 3G). The levels of all three proteins remained equal in the input extracts (Fig. 3H). These results are entirely in agreement with the SILAC data. We also tested whether the reduction in RBPs after MMS treatment was due to formation of higher order complexes shielding them from UVC-induced cross-linking, by increasing the UVC dose from 25 to 100 mJ/cm^2^. As seen in Fig. 3I (lanes 2–4), increasing the dosage did not increase the amount of RBPs. Thus, there was no evidence of bias caused by differential RBP cross-linking after stress.

The low number of proteins with significantly altered mRNA binding after MMS treatment was somewhat surprising. A potential explanation could be that MMS likely mediates very low stoichiometry in the modification at individual sequence sites. It might then be assumed that proteins displaying differential mRNA binding immediately after MMS treatment represent proteins that directly interact with the methylated bases and have little dependence on sequence context, or that belong to RNA metabolic events directly triggered by such recognition.

### Reduced mRNA binding of 40S ribosomal subunits supports blocked 5ʹ-entry of 43S preinitiation complexes

**Fig. 4:**
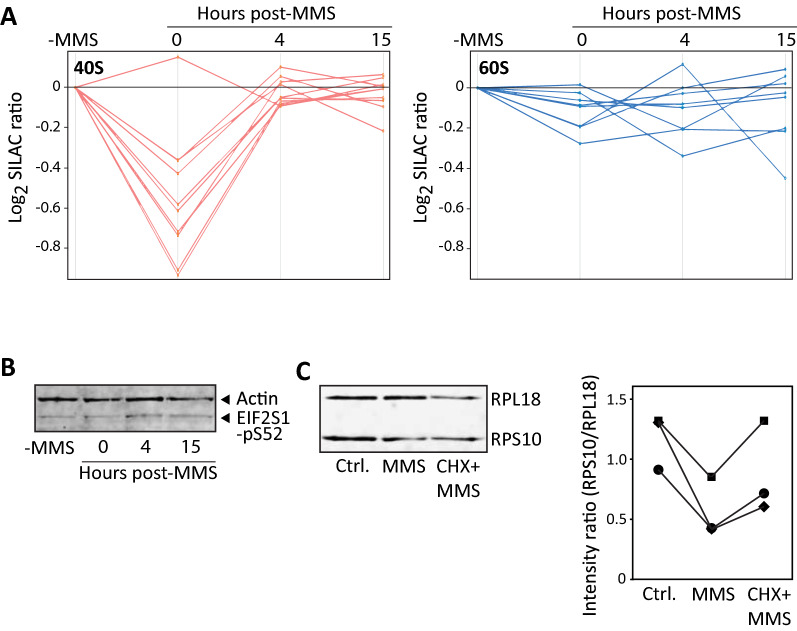
Selective loss of 40S subunits from MMS-treated mRNA. **A** Five out of ten 40S subunits showed statistically significant transiently reduced mRNA binding after MMS treatment, whereas additional four 40S subunits demonstrated very similar, although sub-significantly altered binding pattern (left panel). Such transiently reduced binding was not observed with the 60S subunits (right panel). **B** 1 h MMS-treatment mediated negligible change in the level of phosphorylated (S52) EIF2S1 within 15 h after treatment (normalized to actin). **C** Western analysis (left panel) of mRNA-binding proteins after oligo(dT) capture confirmed a marked decrease in the ratio of bound 40S (RPS10) versus 60S (RPL18) subunits in agreement with the SILAC results. Notably, treatment with cycloheximide (10 μg/ml) for 15 min prior to the MMS treatment (CHX) to decrease ribosomal collision, partially or fully restored the RPS10/RPS18 ratio. The right panel shows RPS10/RPL18 ratios bound to mRNA in three independent experiments (given as three individual graphs) after western blotting and probing with IRDye® secondary antibodies and demonstrating the same trend (Spearman rank correlation = 1 between the three series)

Of the proteins displaying significantly reduced mRNA binding immediately after MMS treatment, several were subunits of the small (40S) ribosomal subunit (Table 1). In addition, the binding of all except one of the other 40S subunits in our dataset was reduced immediately after MMS treatment, although non-significantly according to our criteria (Additional file 4: Table S1). These 40S subunits displayed the same binding pattern during the time course of the experiment, returning to control levels at 4 h after MMS (Fig. 4A left panel, Additional file 4: Table S1), strongly supporting that MMS mediates transient loss of the 40S subunit from the captured mRNA. Conversely, none of the eight large ribosomal subunits in our dataset displayed significantly altered mRNA binding after MMS (Fig. 4A right panel, Additional file 4: Table S1). There is currently no experimental evidence suggesting that 40S ribosomal subunits may be lost from intact ribosomes while retaining the 60S subunit attached to the mRNA. Thus, it is more likely that the MMS treatment mediates reduced loading and accumulation of 43S preinitiation complexes (PICs) at the 5ʹ-UTR. Several forms of cell stress result in an integrated stress response mediated by phosphorylation of the guanine exchange factor EIF2S1 [68], thereby inhibiting formation of 43S PICs. To test this, we quantified phosphorylated EIF2S1 in untreated HeLa cells and at varying time points after 1 h MMS exposure. As shown in Fig. 4B, MMS-treatment mediated negligible change in EIF2S1 phosphorylation within 15 h after MMS treatment, rendering reduced PIC accumulation via this pathway less likely. Notably, two recent studies identified an alternative pathway to block translation initiation and that is independent of EIF2S1 phosphorylation. Instead, ribosomal collisions trigger recruitment of GIGYF2 and 4EHP, which sequester the mRNA cap and blocks recruitment of 43S PICs [39, 40]. To investigate this, we treated the cells with cycloheximide prior to MMS treatment. Cycloheximide arrests ribosomal movement after one translocation step [69] and would thus obstruct ribosomal collisions. Whereas MMS treatment alone mediated a marked loss in the ratio of RPS10 to RPL18, the loss was reversed to varying degrees by cycloheximide prior to the MMS treatment (Fig. 4C, right panel). This supports that the MMS-induced lesions promote RQC-associated arrest of translation initiation at affected mRNAs, thereby mediating the reduced 40S/60S ratio. Potentially, cycloheximide treatment could also block RQC-associated no-go decay (NGD). In yeast, endonucleolytic cleavage occurs at persistently collided ribosomes [70]. This would lead to selective loss of the 5’-UTR-containing fragment and accumulated 43S PICs during oligo(dT) enrichment. However, in human cells mRNA degradation is apparently less associated with RQC than in yeast [71].

The most downregulated protein immediately after MMS treatment was SERBP1, a multifunctional RNA binding protein associated with the 40S ribosomal subunit and actively translating ribosomes [72]. This agrees with a transiently reduced recruitment of 43S PICs. ZC3HAV1 (ZAP), and SND 1 also displayed significantly reduced binding immediately after MMS treatment (Table 1). ZC3HAV1has been shown to target mRNA translation by interfering with the translation initiation complex [73] whereas SND1 is a multifunctional protein that recently was demonstrated to be a *bona fide* m^6^A-binding protein [74]. It is a known binder of metadherin (MTDH), a dsRNA binder and proposed oncogene overexpressed in many cancers [75]. MTDH displayed a similar mRNA binding pattern as SND1 but was quantified in only in only two out of three experiments (1.7- and 2.2-fold reduced binding immediately after MMS treatment) and thus reported as non-significant. This also holds true for the de-ubiquitinase USP10, which displayed 1.8- and 2.7-fold reduced binding immediately after treatment. Recent PAR-CLIP analyses demonstrated that USP10 is enriched at the CDS and that it rescues mono-ubiquitinated 40S subunits subsequent to ribosomal collision and splitting, from otherwise programmed lysosomal degradation [76].

### MMS mediates increased mRNA binding of proteins associated with methylated RNA bases

ANOVA analysis identified that the m^5^C methyltransferase NSUN2 differentially bound to mRNA after MMS treatment, displaying 1.5-fold increased binding after 15 h (Table 1). NSUN2-mediated deposition of m^5^C on mRNA is induced by stress in plants [77], whereas in mammals NSUN2 modulates protein synthesis subsequent to stress by forming m^5^C in tRNAs [78, 79]. Since m^5^C is not directly induced by MMS (Fig. 1), it is tempting to speculate that the increased binding reflects increased NSUN2 targeting to mRNA as part of an overall stress response.

Using our stringent criteria, no proteins displayed statistically significant increased binding to mRNA immediately after MMS treatment. However, manual inspection of the dataset identified four proteins that were readily identified immediately after the treatment, but that were barely detectable at later time points or in the control. This led to large variances in their SILAC-ratios (Additional file 4: Table S1) contributing to making their p-values non-significant [80]. These were gem-associated protein 5 (GEMIN5), probable ATP-dependent RNA helicase and m^6^A reader YTHDC2, the E3 ligase/ISG15 ligase TRIM25 and the activating signal cointegrator 1 complex (ASCC) subunit 3 (ASCC3) (Table 1). GEMIN5 is a 169 kDa multidomain protein that is part of the SMN complex that is essential for spliceosome formation. In addition, GEMIN5 has a key role in reprogramming cellular translation. It can bind to the m^7^G cap and the large ribosomal subunit and is able to mediate global translational repression while enhancing translation of mRNAs harboring thermodynamically stable secondary structure motifs [81]. To what degree GEMIN5 recognizes internal m^7^G or secondary mRNA structures induced by methylation remains, however, to be investigated. The RNA-dependent E3 ubiquitin ligase TRIM25 plays an important role in innate immunity, but the mechanisms underlying this are still poorly understood ([82] and references therein). Very recently, TRIM25 was shown to ubiquitinylate the m^6^A readers IGF2BP1/2/3 and mediate their degradation. This was promoted by binding of the IGF2BPs to m^6^A-modified circular RNA *circNDUFB2* and was dependent on the RNA-binding motif in TRIM25 [83]. To what extent methylated ribonucleosides modulates RNA binding and E3 ligase activity of TRIM25 itself is unknown, but such studies are warranted given known strategies used by viruses to modulate their RNA methylation pattern to avoid host immune responses [84]. The 3ʹ–5ʹ RNA helicase YTHDC2 is an m^6^A reader that is essential for male and female fertility in mice [85]. It has also been found to associate with the 40S ribosomal subunit as well with XRN1, which has a role in degradation of polyadenylated NGD fragments [86].

The increased binding of ASCC3 after MMS treatment was especially interesting. In addition to its role in the ASCC complex [87], ASCC3 was very recently also shown to be part of the human RQT complex, which recognizes stalled ribosomes during mRNA translation to induce subunit dissociation and facilitate RQC [43]. In the nucleus, ASCC3 binds directly to ALKBH3 and stimulates DNA demethylation of m^3^C and m^1^A via its DNA helicase activity. Knockdown of either ALKBH3 or ASCC3 significantly increased sensitivity towards MMS [41]. Since we previously demonstrated that ALKBH3 demethylates RNA with about the same efficiency as ssDNA [33], we speculated whether ASCC3 could mediate a similar stimulatory effect upon demethylation of RNA as of DNA, e.g. by recruiting ALKBH3 or alleviate steric hindrance to demethylation by stalled ribosomes.

### ***ASCC3 contributes to m***^***1***^***A and m***^***3***^***C sanitation from mRNA following MMS treatment***

**Fig. 5:**
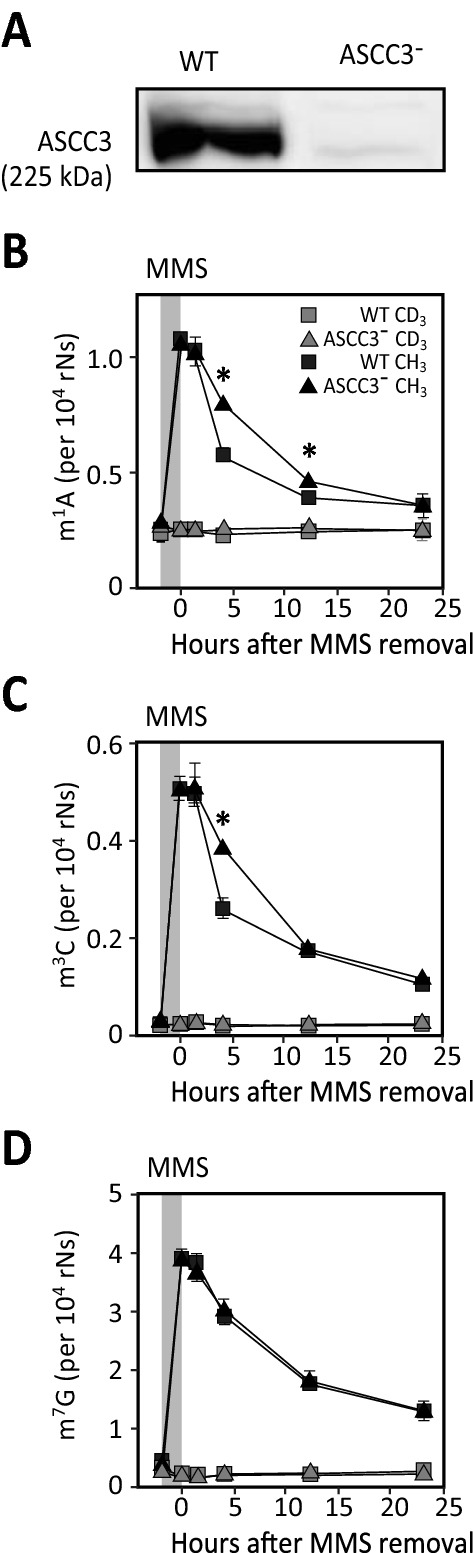
Knockdown of ASCC3 mediates delayed removal of aberrant m^1^A and m^3^C, but not m^7^G, from the mRNA pool. PC-3 prostate cancer cells were subjected to stable lentiviral knockdown of ASCC3. Knockdown and wild type (WT) cells were treated with 1 mM MMS for 1 h (light grey field) and mRNA extracted at various time points. Each data point represents the mean of three biological replicates with SDs as indicated. Removal of MMS-induced (CH_3_) m^1^A (**A**) and m^3^C (**B**) was significantly reduced in the ASCC3-deficient cells compared to WT at 4 h and 12 h (m^1^A) and 4 h (m^3^C), respectively (* t-test, p < 0.001), whereas no such effect was observed for removal of m^7^G (**C**)

To investigate whether ASCC3 influences removal of aberrant methylbases from the mRNA pool, WT and ASCC3-deficient PC-3 prostate cancer cells (Fig. 5A) were subjected to MMS treatment for 1 h and the levels of m^1^A, m^3^C and m^7^G in mRNA were quantified in at various time points. In agreement with the analyses in HeLa cells (Fig. 1), MMS-treatment did not mediate a significant change in the enzymatically induced methylbases (CD_3_), whereas a strong induction of chemical methylation (CH_3_) was observed. Notably, significantly delayed removal of m^1^A (4 h and 12 h, Fig. 5B) and m^3^C (4 h Fig. 5C) was observed after MMS treatment of the ASCC3-deficient cells compared to controls, whereas no such delay was observed for m^7^G (Fig. 5D). This supports a function of ASCC3 in promoting selective removal of aberrant m^1^A and m^3^C from the mRNA pool and suggests that ASCC3 promotes discrimination between the MMS-induced and endogenous methylations.

### ASCC3 promotes assembly of MMS-induced, ALKBH3-containing P-bodies

**Fig. 6:**
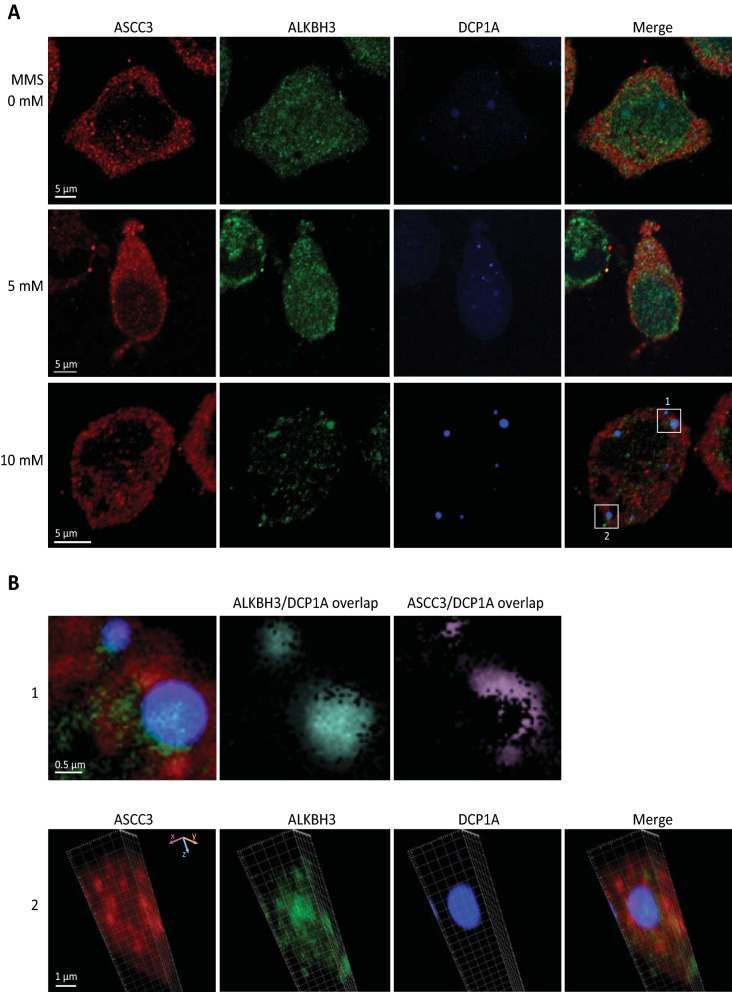
MMS-induced P-bodies in HeLa are enriched in ALKBH3 and localized adjacent to ASCC3. **A** HeLa cells transfected with P-body marker CFP-DCP1A were treated for 1 h as indicated, fixed, and double stained with anti-ASCC3 (red) and anti-ALKBH3 (green). **B** Enlarged images from white squares in **A** showing overlap of DCP1A and ALKBH3, and accumulation of ASCC3 at the P-body periphery (upper panel, corresponding to section 1). The lower panel shows z-stacks of section 2 demonstrating that ASCC3 accumulates around MMS-induced P-bodies whereas ALKBH3 accumulates inside the same P-bodies (lower panel)

To further study a potential role of ASCC3 in recognition- and processing of methylated mRNA we investigated to what degree ASCC3 associated with known cellular ribonucleoprotein structures. We recently demonstrated that ASCC3 predominantly localizes to nuclei both in untreated and MMS-treated cells [42]. To avoid potential leakage of cytoplasmic factors caused by the nuclear permeabilization- and washing steps in the previous study, we reduced the concentration of Triton X-100 from 0.2 to 0.1% and omitted the use of NP-40. This resulted in a clear cytoplasmic staining of endogenous ASCC3 in HeLa cells, forming a dense pattern of diffuse foci throughout the cytoplasm and fewer foci in the nucleus (Fig. 6A, upper panel). A similar, but less dense pattern of ALKBH3-staining was observed throughout the cells, but we could not detect evident focal co-localization of ASCC3 and ALKBH3. Treatment of the cells with 5 mM MMS for 1 h mediated little change in distribution of either ASCC3 or ALKBH3 (Fig. 6A, middle panel). To investigate whether MMS-treatment mediated re-localization to stress-inducible cytoplasmic RNP compartments, we used antibodies against the stress granule (SG) marker TIA1, and transfection with a vector encoding the P-body marker mRNA-decapping enzyme 1A (DCP1A). MMS treatment did not induce stress granules in the cells (data not shown), in agreement with previous findings [88]. Conversely, large and diffuse P-bodies were present in the nucleus both without and after 5 mM MMS-treatment compared to smaller and more sharply defined P-bodies in the cytoplasm, especially after 5 mM MMS treatment. Aggregation of large, sharply defined cytoplasmic P-bodies were observed after an elevated (10 mM) dose of MMS (Fig. 6A, bottom panel). These MMS-induced P-bodies were generally enriched in ALKBH3 (Fig. 6B), whereas ASCC3 was often enriched around the P-bodies. This was most evident from x/y cross-sectional images as illustrated in Fig. 6B (bottom panel).

**Fig. 7:**
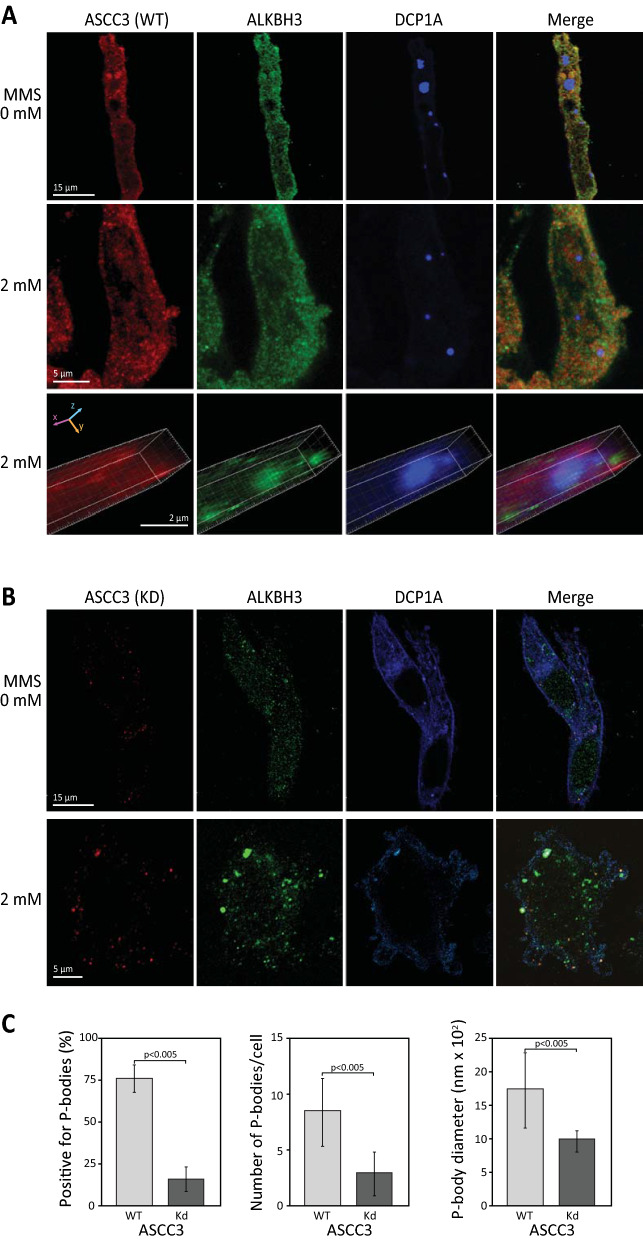
ASCC3 promotes formation of P-bodies in PC-3 cells. PC-3 cells transfected with P-body marker CFP-DCP1A were MMS-treated for 1 h as indicated, fixed, and double stained with anti-ASCC3 (red) and anti-ALKBH3 (green). **A** PC-3 cells proficient in ASCC3 contain P-bodies both in the absence (upper panel) and presence (middle panel) of MMS-treatment. The bottom panel shows enlarged z-stack demonstrating co-localization of DCP1A and ALKBH3, whereas the P-body interior contains less ASCC3 than the volume immediately surrounding the same P-body. **B** Formation of P-bodies was severely impeded in ASCC3 knockdown PC-3 cells both in the presence and absence of MMS. **C** Quantitative analysis of P-bodies in WT and ASCC3 knockdown (Kd) PC-3 after 2 mM MMS treatment demonstrated that significantly fewer ASCC3-deficient cells contained P-bodies compared to WT. In the cells that contained P-bodies, these were also significantly fewer and smaller than in the parental cells

In parental PC-3 prostate cancer cells, a similar staining pattern of ASCC3 and ALKBH3 was observed as in HeLa, except that more distinct cytoplasmic P-bodies were present in the untreated PC-3 cells (Fig. 7A, upper panel). In PC-3 cells treated with 2 mM MMS, ALKBH3 accumulated within P-bodies whereas little ASCC3 was seen in the interior volume of the cytoplasmic P-bodies compared to the surrounding volume, similar to MMS-treated HeLa cells (Fig. 7A, middle and bottom panels). In the ASCC3-deficient cells the ASCC3 strongly reduced and limited to distinct small foci of which many co-localized with ALKBH3 (Fig. 7B, upper panel). This verified efficient depletion of ASCC3 as well as specificity of the antibody. MMS-treatment of the ASCC3-deficient PC-3 cells mediated redistribution of ALKBH3 into fewer and enlarged foci, similar to that observed in HeLa, and of which several overlapped with ASCC3 (Fig. 7B, bottom panel). Strikingly, we found a marked reduction in the fraction of ASCC3-deficient cells positive for P-bodies, and the number and size of P-bodies present in these cells (Fig. 7B,C). This strongly suggests that ASCC3 is involved in the formation of MMS-induced P-bodies but does not constitute a prominent part of the P-body itself.

## Discussion

Here we show that the methylating agent MMS induces direct formation of m^1^A, m^3^C and m^7^G in human poly(A)-enriched RNA at levels considerably exceeding those in total RNA, while the overall levels of the corresponding endogenous modifications remain essentially unaffected.

Somewhat surprisingly, the aberrant methylbases induced by MMS did not mediate prominent skewing of the mRNA binding proteome. Thermodynamic dissociation constants (*K*_D_) so far measured for proteins able to bind modified mRNAs suggest that the modifications mediate modestly (2- to 20-fold) altered mRNA binding [1]. Moreover, binding of individual proteins is also affected by local sequence context, and thus altered binding of specific RBPs will likely affect only small subsets of mRNAs. Translating ribosomes constitutes an exception to this by offering a means of universal quality control of coding RNA, and ribosomal collisions signal potentially defective mRNA. RQC has been shown to be activated in response to MMS and oxidizing agents in yeast, and mutants defective in RQC components recover poorly subsequent to treatment [89]. Recent studies in yeast [70] and mammalian cells [90] demonstrated that the 40S–40S interface at persistently stalled and collided ribosomes are detected by ZNF598 (Hel2 in yeast), which ubiquitinylates the leading 40S subunit and mediates recruitment of the ASCC complex containing ASCC3 (Slh1 in yeast). The helicase activity of ASCC3 is essential for splitting of the leading ribosome [90]. Very likely, this increases accessibility of ALKBH3 to demethylate translation-blocking m^1^A and m^3^C buried within the decoding center of the leading ribosome. Independent of its ubiquitin ligase activity, ZNF598 also mediates formation of a ZNF598-GIGYF2-4EHP complex at collided ribosomes. Here, 4EHP (EIF4E2) outcompetes binding of eIF4E to the 5’-cap of affected transcripts, thereby inhibiting translation initiation by blocking access of 43S PICs [39, 40]. Since multiple PICs are bound at the 5’-UTR [91–93] this would lead to selective loss of 40S subunits after MMS treatment (Fig. 4D). In yeast, Hel2 and Slh1 were both crucial for RQC as well as for degradation of affected transcripts by NGD. Endonucleolytic cleavage occurred in the region spanning the leading stalled ribosome and the colliding ribosome [70], and the 5ʹ- and 3ʹ-fragments are degraded by the exosome (stimulated by Dom34:Hbs1) and the 5ʹ–3ʹ-exonuclease Xrn1, respectively [94]. In human cells, activation of the RQC pathway appears to be less accompanied by mRNA degradation compared to yeast [71]. Nevertheless, it cannot be ruled out that NGD contributes to the observed loss of 40S subunits after MMS-treatment, since mRNA cleavage would result in loss of the 5’-UTR during oligo(dT)-mediated capture and loss of associated 43 PICs.

Several lines of evidence support a function of ASCC3 in promoting removal of MMS-induced m^1^A and m^3^C from the mRNA pool: (i) ASCC3 displays transiently increased mRNA binding subsequent to MMS-treatment, (ii) knockdown of ASCC3 mediates delayed removal of m^1^A and m^3^C, but not m^7^G, from the mRNA pool after MMS treatment, (iii) ASCC3 promotes formation of MMS-induced P-bodies, which are prominent sites of mRNA turnover and (iv) ASCC3 and its yeast homolog Slh1 both couple ribosome arrest to RQC-mediated ribosome stripping and nascent polypeptide degradation [90, 95]. Within the translated pool of mRNA, ASCC3 might serve a dual function in the sanitation of aberrant methylbases: When ribosomes stall at m^1^A or m^3^C it mediates ribosomal splitting and recruits ALKBH3 to catalyze direct demethylation and allow restored translation. When these substrates are located in less accessible secondary structures, prolonged ribosomal stalling may promote transfer of the aberrantly methylated mRNA to P-bodies, similar to the postulated role of YTHDF2 in the transfer of m^6^A-containing mRNA from the translatable pool to RNA decay sites [21]. P-bodies are formed by phase separation largely driven by interaction between proteins rich in lysine-containing disordered regions, and RNA [96]. It is tempting to speculate that ASCC3-mediated stripping of ribosomes, which are highly structured ribonucleoproteins with an overall negative surface [97], is an important step to induce phase separation and formation of P-bodies. This is also supported by studies showing that P-bodies are virtually devoid of ribosomal proteins [98].

The precise contribution of ASCC3/ALKBH3-mediated demethylation versus depletion of m^1^A and m^3^C by RNA degradation cannot be precisely determined from the present study. Nevertheless, some rough estimates can be made from the kinetics of depletion of MMS-induced m^1^A, m^3^C and m^7^G. Mammalian mRNA, which constitutes the bulk of the poly(A)-RNA pool, has an average half-life of 9 h [99]. Thus, the bulk of mRNA harboring aberrant methylations should be degraded by canonical mRNA turnover alone within 24 h after MMS treatment. In addition to the canonical turnover, translated transcripts harboring aberrant methylbases will be subject to ASCC3/ALKBH3-mediated demethylation and potentially also NGD. At early time points (4 h) ASCC3-mediated removal, most likely by ALKBH3-catalyzed demethylation, is responsible for about one-third of the MMS-induced m^1^A and m^3^C lesions (Fig. 5B, C). Based on the virtually identical removal of mRNA containing aberrant m^7^G at early time points after treatment in the ASCC3-proficient cells compared to the removal of m^1^A and m^3^C in the ASCC3-deficient cells (4 h, Fig. 5B–D), aberrant m^7^G is likely removed by canonical RNA turnover and potentially also NGD, since m^7^G may modulate mRNA secondary structure [6] and base pairing of m^7^G with G is often observed in double stranded RNAs [32]. It cannot be excluded, however, that mammalian cells also express a demethylase that contributes to internal m^7^G removal. Interestingly, a recent study found that internal m^7^G is enriched at the 5ʹ-UTR and AG-rich sequences in unstressed cells, whereas increased m^7^G deposition was observed in the CDS and 3ʹ-UTR after H_2_O_2_ and heat shock treatments [7]. Increased m^7^G in the 3ʹ-UTR was accompanied by increased translation efficiency of a minigene reporter and the authors suggest that stress-induced m^7^G might be involved in the signaling pathways for specific stress responses [7].

**Fig. 8:**
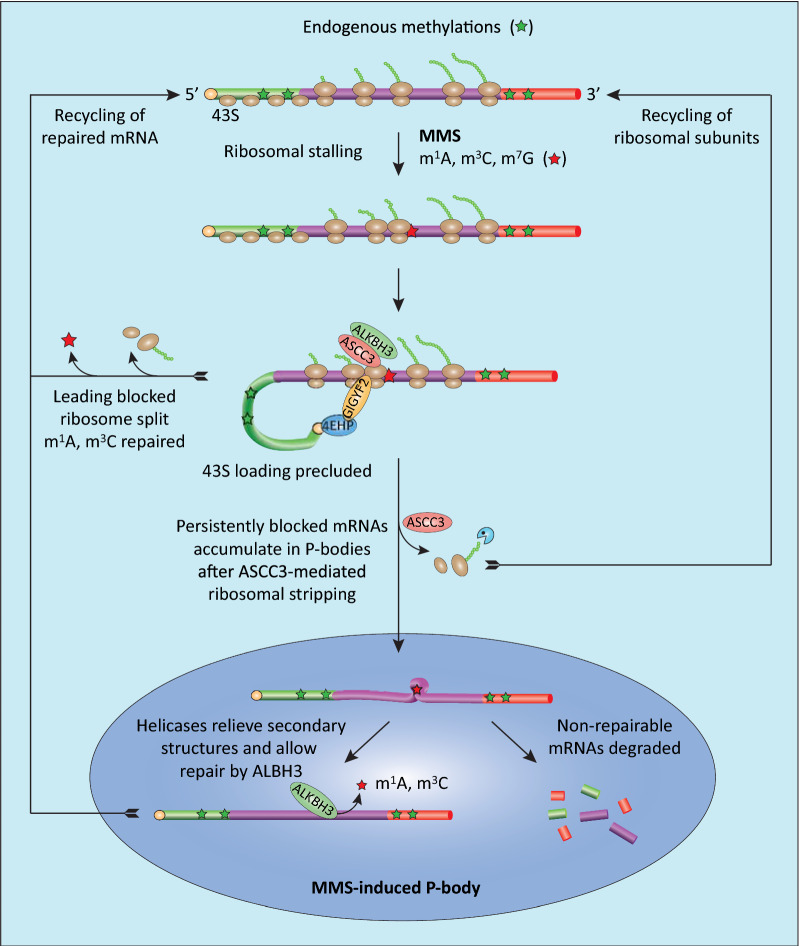
Working model of cellular processing of aberrantly (MMS-) methylated mRNA. MMS-induced m^1^A and m^3^C mediate direct ribosomal stalling, whereas m^7^G may form translation-blocking secondary structures. Collided disomes are detected by ZNF598, which serves two functions: It stabilizes GIGYF2-4EHP to block cap-dependent 43S entry and facilitates recruitment of ASCC3/ALKBH3 to mediate ribosome splitting and removal of m^1^A/m^3^C. Repaired transcripts can then be re-routed directly to translation. Transcripts harboring persistent blocking lesions, e.g. MMS-induced secondary structures, are stripped of ribosomes by ASCC3 and accumulate in P-bodies. Here, helicase activities relieve secondary structures to promote repair by ALBH3, whereas nonrepairable mRNAs are degraded

Based on accumulated experimental evidence and our data, we propose a working model (Fig. 8) in which MMS induces aberrant methylbases that inhibit translation and potentially affect other mRNA functions. In the translated mRNA, aberrant methylbases in the coding region mediate ribosomal stalling and collision. ASCC3 recruits ALKBH3 to the arrested ribosomes, promotes splitting of the first arrested ribosome and renders m^1^A and m^3^C accessible for oxidative demethylation by ALKBH3. mRNAs harboring persistent translational blocks may then accumulate in P-bodies. Prior to accumulation in P-bodies, aberrantly methylated mRNAs and mRNA fragments are stripped of ribosomal subunits, likely via an ASCC3-initiaded RQC pathway involving degradation of partial nascent polypeptides and recycling of ribosomal subunits (analogous to the function of Slh1 in yeast [95]). P-bodies contain the endonucleolytic activities necessary for degradation of the mRNA as well as RNA helicases that may relieve secondary structures and potentially allow accessibility to repairable modifications [100]. After repair, the mRNAs can then be routed back to the translatable pool of mRNAs.

The biological impact of ASCC3/ALKBH3-mediated removal of MMS-induced methylbases from mRNA yet remains to be investigated. Enzymatic demethylation of mRNA would be energetically favorable compared to mRNA degradation and resynthesis and could be especially important during cell stress. Interestingly, ALKBH3 was recently shown to protect against cell stress by demethylating tRNA. This rendered the tRNA susceptible to cleavage by angiogenin, thus producing tRNA-derived small RNAs (tDRs) that strengthened ribosome assembly on mRNA and prevented apoptosis triggered by cytochrome c [101]. It is tempting to speculate that excessive chemical induction of m^1^A and m^3^C in mRNA might negatively affect this defense mechanism by diverging ALKBH3 from tRNA demethylation. Depletion of either ALKBH3 or ASCC3 has previously been shown to reduce survival of several cell lines subsequent to MMS treatment [41]. However, the contribution of hampered DNA repair versus RNA repair/degradation to the increased cytotoxicity must await further studies.

## Conclusion

Our study demonstrates that MMS abundantly induces methylated bases in human mRNA identical to those found endogenously. Our proteomic analyses furthermore highlight potential mechanisms that enable cells to distinguish between canonical and aberrant methylations. In translated mRNA, aberrant methylations in the coding region apparently induces RQC of the affected transcripts. ASCC3 mediates ribosomal splitting and may recruit ALKBH3 to demethylate aberrant m^1^A and m^3^C. ASCC3 also facilitates formation of P-bodies, potentially by stripping ribosomes from transcripts harboring persistent translational blocks. Our findings warrant further studies on the capacity of alkylating agents to mediate (epi)transcriptome dysregulation and the potential contribution of aberrant RNA methylation to the cytotoxic effects of alkylating drugs. These studies should include other RNA species such as tRNAs and rRNAs as well as R-loops, in which altered base methylation patterns are often associated with human cancers [102].

## Supplementary Information

**Additional file 1: Figure S1.** Optimization of UV-C dose for RBP-RNA crosslinking.

**Additional file 2: Figure S2.** Effects of MMS treatment on viability and cell cycle distribution of HeLa cells.

**Additional file 3: Figure S3.** Experimental pipeline of the SILAC analyses.

**Additional file 4: Table S1.** Results from SILAC analysis of RBPs after MMS treatment.

## Data Availability

The proteomics data have been deposited to the ProteomeXchange Consortium via the PRIDE partner repository with the identifier PXD003549 [103].

## Abbreviations

ALKBH3: Alpha-ketoglutarate-dependent dioxygenase alkB homolog 3
ASCC3: Activating signal cointegrator 1 complex subunit 3
CDS: Coding sequence
CFP: Cyan fluorescent protein
DMEM: Dulbecco’s modified Eagle medium
FACS: Fluorescence-activated cell sorting
HA: Hemagglutinin
LC–MS/MS: Liquid chromatography-tandem mass spectrometry
MMS: Methyl methanesulfonate
MNU: 1-Methyl-nitrosourea
NGD: No-go decay
PAR-CLIP: Photoactivatable ribonucleoside-enhanced crosslinking and immunoprecipitation
P-body: Processing body
RBP: RNA binding protein
RPL: Ribosome large (60S) subunit
RPS: Ribosome small (40S) subunit
RQC: Ribosome quality control
RQT: Ribosome quality control trigger
SAM: *S*-Adenosyl methionine
SG: Stress granule
SILAC: Stable Isotope Labeling with Amino acids in Cell culture
UTR: Untranslated region
YTH-domain: YT521-B homology domain
